# Immunomodulatory and antitumour effects of abnormal Savda Munziq on S180 tumour-bearing mice

**DOI:** 10.1186/1472-6882-12-157

**Published:** 2012-09-17

**Authors:** Ainiwaer Aikemu, Anwar Umar, Abdiryim Yusup, Halmurat Upur, Bénédicte Berké, Bernard Bégaud, Nicholas Moore

**Affiliations:** 1 Department of Drug Analysis, Xinjiang Medical University, 830011, Urumqi, Xinjiang, People’s Republic of China; 2 Faculty of Traditional Uyghur Medicine, Xingjiang Medical University, 830011, Urumqi, Xingjiang, People’s Republic of China; 3 Department of Pharmacology, Xinjiang Medical University, 830011, Urumqi, Xingjiang, People’s Republic of China; 4 Department of Pharmacology, Univ Bordeaux, F-33000, Bordeaux, France

## Abstract

**Background:**

Abnormal Savda Munziq (ASMq), a traditional uyghur medicine, has shown anti-tumour properties *in vitro*. This study attempts to confirm these effects *in vivo* and measure effects on the immune system.

**Methods:**

Kunming mice transplanted with Sarcoma 180 cells were treated with ASMq (2–8 g/kg/day) by intra-gastric administration compared to model and cyclophosphamide (20 mg/kg/day). After the 14th day post tumour implant, thymus, liver, spleen and tumours were removed, weighed, and processed for histopathological analysis. Blood samples were also taken for haematological and biochemical analyses including TNF-α , IL-1 β and IL-2. Splenic lymphocyte function was measured with MTT; lymphocyte subpopulations were measured by flow cytometry.

**Results:**

ASMq treated animals had reduced tumour volume compared to model and increased concentrations of TNF-α, IL-1β and IL-2 compared to untreated and to cyclophosphamide-treated animals. No histopathological alterations were observed. The absence of viable S180 cells and the presence of necrotic cells and granulation tissue were observed in tumour tissue of treated animals. The effect on T lymphocytes was unclear.

**Conclusions:**

ASMq confirmed in vivo anti-tumour effects observed in vitro, which may be at least in part mediated by increased immune activity.

## Background

Abnormal Savda Munziq (ASMq), a traditional Uyghur medicinal herbal preparations from the Xinjiang region of China has long been used in Traditional Uyghur Medicine for the treatment of diseases such as digestive cancer, diabetes, cardiovascular diseases or chronic asthma [1]. Uyghur medicine considers Abnormal Savda as a final pathologic product of ”combusted” body fluid (e.g. Savda, Sapra, Khan, Balgham), heavier in weight and thicker in texture, and easy to deposit against the blood vessel thus causing dilatation, which ultimately leads to chronic diseases such as tumour and cancer. Therapy for Abnormal Savda-related diseases is based initially on ASMq, before using other drugs such as Abnormal Savda Mushil. This results in temperament recovery and body fluid equilibrium. ASMq is widely applied traditionally for the treatment of complex diseases such as tumours.

Therefore it would seem appropriate to test the effects of ASMq on the development of tumours in animals, and verify biological data that might confirm effects and orient towards a mechanism of action.

ASMq is a herbal formula that is described in the Chinese Uyghur pharmacopoeia, composed of ten medicinal herbs (Table 1) [2]. It is now produced commercially (patent no. ZL02130082.8). For years, we have been studying its effects, and so far these studies have shown the capacity of ASMq to scavenge free radicals and superoxide anions, [3] decrease biological markers of oxidative stress in man, [4] protect mitochondria and DNA [3] against OH^-^ induced oxidative damage in a cell-free system [1], and inhibiting cancer cells proliferation and viability in vitro [2,5-7] and in vivo [2].

**Table 1:** Plants used in Uyghur herbal preparation Abnormal Savda Munziq (ASMq)

**Latin name**	**Family**	**Part used**	**Uyghur Name**	**Chinese Name**
*Adiantum capillus-veneris* L.	Adiantaceae	Whole plant	Pirsiyavxan	Tiexianjue
*Alhagi pseudalhagi* (Bieb.) Desv.	Fabaceae	Branch secretion	Kök tantak	Citang
*Anchusa italica* Retz.	Boraginaceae	Whole plant	Gavziban	Niushecao
*Cordia dichotoma* G.Forst.	Boraginaceae	Fruit	Serbistan	Pobumuguo
*Euphorbia humifusa* Willd. *Euphorbia maculata* L.	Euphorbiaceae	Whole plant	Yalmankülak	Dijincao
*Foeniculum vulgare* Mill.	Apiaceae	Fruit	Arpabidiyan	Xiaohuixiang
*Glycyrrhiza uralensis* Fisch. ex DC.	Fabaceae	Radix or rhizoma	Qüqük buya	Gancaogen
*Glycyrrhiza inflata* Batalin	Fabaceae	Radix or rhizoma		
*Glycyrrhiza glabra* L.	Fabaceae	Radix or rhizoma		
*Lavandula angustifolia* Mill.	Lamiaceae	Aerial parts	Üstihuddus	Xunyicao
*Melissa officinalis* L.	Lamiaceae	Whole plant	Badrenjiboye hindi	Mifenghua
*Ziziphus jujuba* Mill.	Rhamnaceae	Fruit	Qilan	Dazao

Because immunity plays a major role in protection against cancer, and because ASMq might have an effect also on the immune system, [8] we tested in this study not only the effect of ASMq on tumour growth, but also on markers of immunity such as TNF-α, interleukins IL-1β, or IL-2, and the distribution of T-lymphocytes. The effects of ASMq were compared to those of cyclophosphamide, a standard comparator for S180 sarcoma cells.

## Methods

### Chemicals and reagents

Industrially prepared ASMq granules were provided by Qikang Habo pharmaceutical Co., LTD, Xinjiang (batch number106060). Cyclophosphamide was purchased from HengRui pharmaceutical Co., LTD, JiangSu under SFDA (State Food and Drug Administration) batch H32020857. Mouse IL-1β ELISA, batch number: 56069011; Mouse IL-2 ELISA, batch number: 55708025; Mouse TNF-α ELISA, batch number: 55674004, were purchased from Bender MedSystems Co., Austria.

### Animals and treatment

Kunming SPF mice (4 to 6 weeks old, body wt. 20 ± 2 g) were provided by the Laboratory Animal Centre of the Xinjiang Medical University. The mice were bred under regular laboratory conditions, i.e., room temperature and 12/12-hour light-dark cycle with free access to standard rodent chow and water. The Laboratory Animal Centre of Medicine Animal Care and Use Committee of the Medical University of Xinjiang at Urumqi approved all experimental protocols. One hundred and twenty mice were randomly divided into six groups of 20 animals:

– control group: no intervention, no treatment (normal saline 20 μl/kg orally daily)

– model group: injection of S180 cells, no treatment. (normal saline 20 μl/kg orally daily)

– Low dose ASMq group: S180 cell injection, ASMq 2 g/kg orally daily

– Medium dose ASMq group: S180 cells injection, ASMq 4 g/kg orally daily,

– High dose ASMq group: S180 cells injection, ASMq 8 g/kg orally daily,

– Cyclophosphamide (CY) group: S180 cells injection, CY 20 mg/kg daily injection.

S180 cells were supplied by Laboratory Animal Centre of Xinjiang military hospital.

Except for the control group, 0.2 mL (1 × 10^7^ cells/ml) of seven-day-old S180 ascites was transplanted into the right axilla of the mouse. The whole operation was finished in 30 min.

These treatments were started 24 h after tumour inoculation and given once a day for 14 days.

### Animal observation

Animals were observed and graded daily for activity, thinness, appearance of skin and hair, appetite, irritability.

Tumour size was measured daily using a ruler, and tumour size was plotted against time to measure tumour growth velocity.

### Assessment of tumour weight, thymus, liver and spleen index

Twenty-four hours after the last treatment administration at day 14, the mice were sacrificed by cervical dislocation. The thymus, spleen, liver and solid tumours were removed and weighed. The anticancer activity in vivo was expressed as an inhibitory rate calculated by the formula: [(A–B)/A] × 100%, where, A and B were the mean tumour weights of the model control and experimental groups, respectively. The spleen, liver and thymus were evaluated by the organ index formula: spleen, liver or thymus weight (g)/body weight (g).

### Histological Investigation

A portion of tumour, kidneys, thymus and liver were fixed in 10% buffered formalin and the remaining tissue was used for biochemical measurements. The tissues were fixed in solutions of ethanol 70% for 3 h, ethanol 80% 2 h, ethanol 90% 1.5 h, ethanol 95% 2 h, and ethanol 100% 1 h. Tissues were embedded in paraffin and at least four cross-sections were taken from each tumour, kidneys, thymus and liver in 4–5 μm thickness and stained with haematoxylin-eosin (H & E). Two changes each of 2 min of xylene treatment were done and finally tissue sections were mounted with DPX mountant. The slides were observed for histopathological changes and microphotographs were taken using an Olympus BX50 microscope system (Olympus, Japan).

### Splenic lymphocytes function

#### MTT test

The viability of the spleen lymphocyte was assessed by MTT (3,4,5-dimethylthiazol-2-yl)-2-5-diphenyltetrazolium bromide) assay, which is based on the reduction of MTT by the mitochondrial dehydrogenase of intact cells to a purple formazan product [5-7,9]. A suspension of spleen cells was diluted to 1 × 10^7^ cell/L; 100 μl of cell suspension was seeded in 96-well plates (Coastar, Corning, NY) with 100 μl RPMI-1640 containing 5 μg/mL ConA, with three wells per sample. These were incubated for 12 h, 48, and 72 h in a 5% CO_2_ incubator at 37 °C. and MTT assays were performed with thymus T lymphocyte. 100 *μ*L of 0.5 mg/mL MTT in cell culture medium was added to each well and incubated for 2 h. 100 *μ*L of 10% SDS, 0.01 M HCl solution was added to each well to dissolve the formazan crystals formed. The plates were covered with aluminium foil and kept in an incubator for 12 h for dissolution of the formed formazan crystals. Amount of formazan was determined measuring the absorbance at 560 nm using a microplate reader (DYNATECH MR 4000).

### Detection of lymphocyte subpopulations

Blood was immediately collected on heparin from the orbital venous plexus after the 14th day treatment. CD_3_, CD_4_, CD_8_ cells and CD_4_/CD_8_ ratio were assessed by flow cytometers (FCM), using PE-Cy^TM^5 Hamster Anti-Mouse CD3e, Batch number 553065;PE Rat Anti-Mouse CD_4_,Batch number 553048;FITC Rat Anti-Mouse CD8a, Batch number 553030. These kits were purchased from BD Biosciences and were used according to the BD Biosciences reagent instructions.

### Evaluation of cytokines

Blood was immediately transferred into test tubes, and kept at room temperature for 30 minutes, according to the manufacturer’s protocol. The blood was then centrifuged at 3000 rpm for 20 min, followed by serum assays for tumour necrosis factor-α (TNF-α); interleukin-1β (IL-1β); interleukin −2 (IL-2) using ELISA methods (see above).

### Statistical procedures

Data are expressed as mean ± SD and were analysed with SPSS 17.0 statistical software using the statistical tests appropriate to the variables. A p value less than 0.05 was considered statistically significant.

## Results

### Mice daily activities

We observed mice behaviour, autonomic activities, ingestion, drinking, hairs, faeces and urine daily. There was no secretion in eye, ear, nose and mouth. The 2 g/kg ASMq group showed normal activity, general mental state, clean fur; tumour growth incubation period (TT) was 3 days, tumour growth velocity (TS) was slower than in the model group. ASMq 4 g/kg group showed normal activity, good mental state (better than 2 g/kg group), clean fur (better than 2 g/kg group); TT was 3d, TS was slower than 2 g/kg group. ASMq 8 g/kg group showed normal activity, good mental state (better than 2 g/kg group and 4 g/kg group), clean fur (better than 2 g/kg group and 4 g/kg group); TT was 3d, and TS was slower than 2 g/kg and 4 g/kg groups. CY group showed normal activity, good mental state (better than 2 g/kg group), clean fur (better than 2 g/kg group); TT was 4d, and TS was the slowest. Model group showed decreased activity, enlarged tumour, gloomy and unclean fur, and listlessness; TT was 2d, and TS was the fastest.

### Treatment effects on growth rate of transplanted S180 tumours and organ weight ratios

The anticancer effects of ASMq against S180 are shown in Table 2 and Table 3. Treatment with cyclophosphamide and ASMq resulted in markedly lower tumour weight. Compared with controls, the tumour inhibition rates in high, medium, low dose ASMq and cyclophosphamide-treated mice were 49.1%, 64.3%, 43.4%, and 50.9%, respectively. Low ASMq dosage group had significantly higher tumour weight than the cyclophosphamide group (*p* < 0.05). The medium-dose ASMq group had lowest tumour weight. High ASMq dose group was not different from cyclophosphamide.

**Table 2:** Effect of treatment with Cyclophosphamide or abnormal Savda Munziq traditional Uyghur Medicine in Mice Transplanted with S180 Tumour on total body and organ weights (mean ± SD)

**Group**	**Dosage (g/kg)**	**Tumour weight (g)**	**Thymus weight (g)**	**Spleen weight (g)**	**Liver weight (g)**	**Body weight (g)**
Control	0	0.00 ± 0.00	0.10 ± 0.01	0.09 ± 0.01	1.21 ± 0.05	30.40 ± 1.44
Model	0	0.60 ± 0.03	0.09 ± 0.02	0.12 ± 0.01	1.30 ± 0.07	32.57 ± 1.50
CY	0.02	0.29 ± 0.01*	0.07 ± 0.01	0.08 ± 0.01	1.15 ± 0.05	25.96 ± 1.32
ASMq low	2	0.34 ± 0.02*^#^	0.10 ± 0.01^#^	0.11 ± 0.01*^#^	1.35 ± 0.13*^#^	27.34 ± 1.26*^#^
ASMq medium	4	0.21 ± 0.18*^#^	0.09 ± 0.01*^#^	0.12 ± 0.02*^#^	1.35 ± 0.37	29.82 ± 2.24^#^
ASMq high	8	0.30 ± 0.53*	0.11 ± 0.14^#^	0.11 ± 0.11*^#^	1.23 ± 1.30	30.22 ± 1.93^#^

* different from the tumour control group, p 0.05;

# different from the CY group, ^#^p 0.05;

CY: cyclophosphamide.

ASMq: abnormal Savda Munziq traditional Uyghur medicine.

**Table 3:** Effect of treatment with Cyclophosphamide or abnormal Savda Munziq traditional Uyghur Medicine in Mice Transplanted with S180 Tumour on organ weight indices (organ weight in mg/bodyweight in g (x ± SD))

**Group**	**Dosage (g/kg)**	**Thymus weight / Mouse weight (mg/g)**	**Spleen weight / Mouse weight (mg/g)**	**Liver weight / Mouse weight (mg/g)**	**Tumour Inhibition (%)**
Control group	0	3.3 ± 0.5	3.1 ± 0.4	39.9 ± 2.8	0
Model group	0	2.8 ± 0.9	3.6 ± 0.4	40.1 ± 2.9	0
CY	0.02	2.6 ± 0.3	2.9 ± 0.4	44.3 ± 3.0	50.88
ASMq low	2	3.6 ± 0.4^#^	4.0 ± 0.5*^#^	49.4 ± 6.0*^#^	43.43
ASMq medium	4	3.0 ± 0.7	4.2 ± 1.0*^#^	46.8 ± 13.2	64.26
ASMq high	8	3.5 ± 0/7^#^	3.5 ± 0.3*^#^	40.8 ± 4.4	49.05

* different from the tumour control group, *p<0.05;

# different from the CY group, #p<0.05.

CY: cyclophosphamide.

ASMq: abnormal Savda Munziq traditional Uyghur medicine.

The spleen index in all ASMq dose groups was significantly higher than that of the model control group or the CY treated mice (Table 3). There was no difference in thymus weight index, but the liver index was higher in low-dose ASMq mice than in model mice.

### Treatment Effects on IL-1β, IL-2 and TNF-α

Effects of ASMq on IL-1β, IL-2, and TNF-α concentrations are shown in Table 4. IL-1, IL-2 and TNF-α concentrations were lower in the model and CY groups than in the control group. Animals treated with ASMq 2 g/kg, 4 g/kg, 8 g/kg had significantly higher TNF-α concentration (*p* < 0.05) than controls but not IL-1β. Compared with the model group, groups treated with ASMq 2 g/kg, 4 g/kg, or 8 g/kg had significantly higher IL-1, IL-2 and TNF-α concentrations.

**Table 4:** Effect of treatment with Cyclophosphamide or abnormal Savda Munziq traditional Uyghur Medicine in Mice Transplanted with S180 Tumour on IL-1β; IL-2; TNF-α in mice blood serum (mean ± SD)

**Group**	**Dosage (g/kg)**	**IL-1β (pg/ml)**	**IL-2 (pg/ml)**	**TNF-α (pg/ml)**
Control	0	511.42 ± 6.06*#	17.64 ± 0.99*#	16.64 ± 0.86*#
Model	0	405.79 ± 7.03	15.23 ± 0.86	14.75 ± 0.67
CY	0.02	447.58 ± 3.38	12.50 ± 0.92	13.53 ± 0.66
ASMq low	2	430.50 ± 4.63*^#^	17.15 ± 1.01*^#^	18.52 ± 0.80*^#^
ASMq medium	4	510.32 ± 3.60*^#^	23.55 ± 1.20*^#^	27.13 ± 0.87*^#^
ASMq high	8	420.03 ± 3.06*^#^	19.99 ± 1.27*^#^	21.30 ± 0.87*^#^

* different from the Model group, *p<0.05;

# different from the CY group, #p<0.05.

CY: cyclophosphamide.

ASMq: abnormal Savda Munziq traditional Uyghur medicine.

### CD_3_^+^, CD_4_^+^ , CD_8_^+^ and CD_4_/CD_8_ Ratio

CD_3_ and CD_4_^+^ cells were lower in all treatment groups than in the control group, CD_3_ were significantly lower in the high ASMq dose and 2 g/kg ASMq groups than in the untreated cancer model (*p* <0.05), a pattern also reproduced for CD4 cells, though not significantly. Only the high dosage group had significantly lower CD_8_^+^ (*p* <0.05) (Table 5).

**Table 5:** **Effect of treatment with Cyclophosphamide or abnormal Savda Munziq traditional Uyghur Medicine in Mice Transplanted with S180 Tumour, on the percentage of CD**_**3**_^**+**^**, CD**_**4**_^**+**^**, CD**_**8**_^**+**^**and CD**_**4**_**/CD**_**8**_**ratio (m ± SD)**

**Group**	**Dosage g/kg)**	**CD**_**3**_^**+**^**T cell (%)**	**CD**_**4**_^**+**^**T cell (%)**	**CD**_**8**_^**+**^**T cell (%)**	**CD**_**4**_**/CD**_**8**_**ratio**
Control	0	60.0 ± 9.6	43.4 ± 10.1	22.9 ± 2.9	1.88 ± 0.30
Model	0	54.6 ± 9.0	37.7 ± 9.7	23.6 ± 5.8	1.62 ± 0.31
CY	0.02	53.4 ± 9.1	39.6 ± 7.6	22.0 ± 3.4	1.80 ± 0.25
ASMq	2	49.0 ± 10.9*^#^	28.3 ± 8.7	19.2 ± 5.8	1.52 ± 0.37
ASMq	4	58.2 ± 16.3	38.0 ± 9.0	21.1 ± 6.0	1.85 ± 0.38
ASMq	8	45.1 ± 7.8*^#^	28.6 ± 7.5	14.7 ± 3.0*^#^	1.85 ± 0.49

* different from the tumour control group, *p<0.05;

# different from the CY group, #p<0.05

CY: cyclophosphamide.

ASMq: abnormal Savda Munziq traditional Uyghur medicine.

### Pathological examination

**Figure 1:**
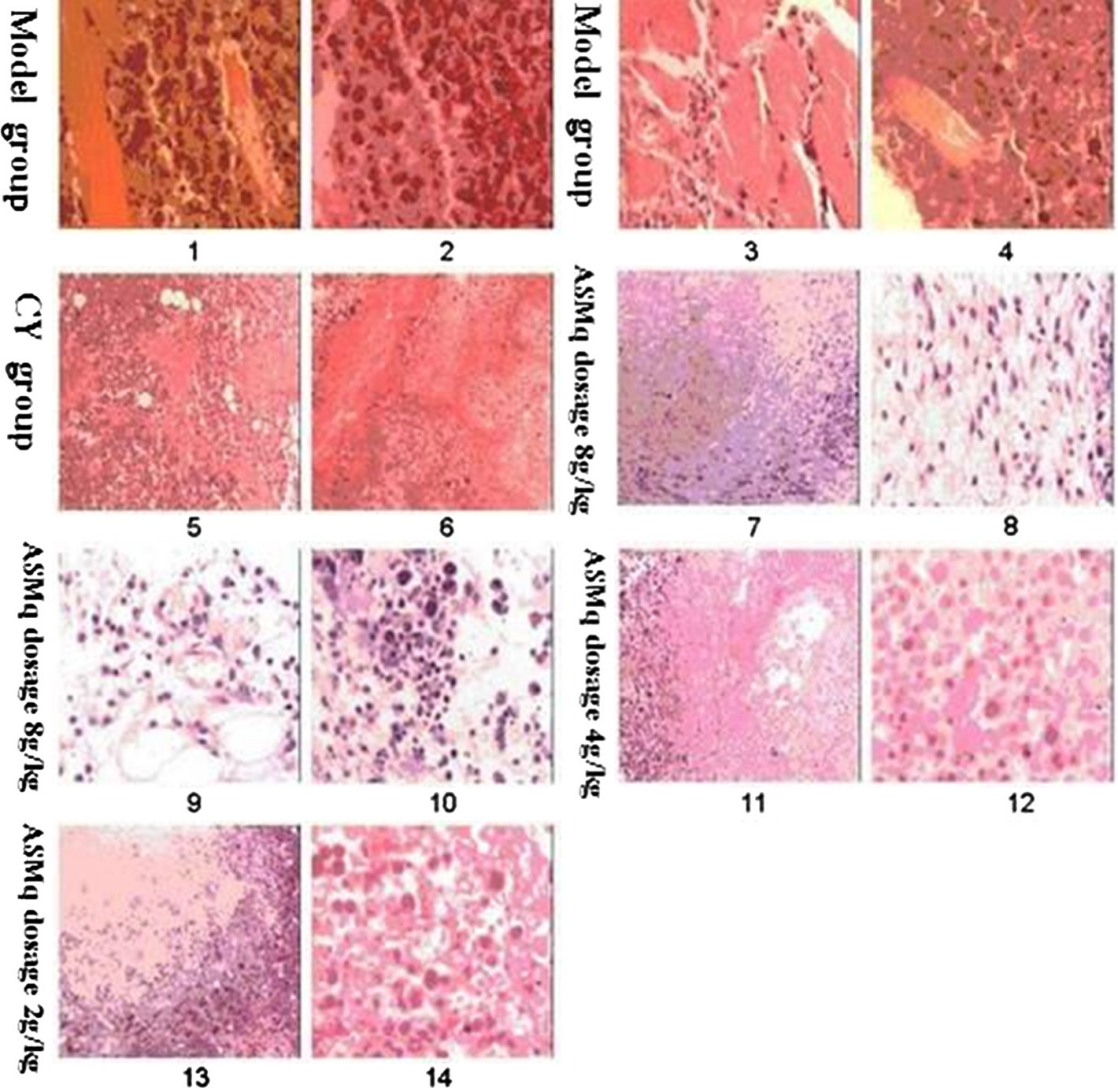
**Histological sections of S180 myosarcoma in Kunming mice.** Photomicrographs of five micron thick histological sections of S180 myosarcoma in Kunming mice: 1–4 untreated controls; 5,6: cyclophosphamide; 7–10: ASMq 8 g/kg; 11,12: ASMq 4 g/kg; 13, 14: ASMq 2 g/kg.

In the untreated Model group, (Figure 1–1 to 1–4) S180 axillary tumours had large, irregular tumour cells, with large nuclei and little cytoplasm. Tumour growth was rapid and invasive, with poorly defined boundaries between tumour and adipose tissues, In Figure 1–1 and 1–3, tumour tissue has invaded the muscle tissue. Although there was little inflammatory infiltrate, the tumoural necrotic areas were very pronounced.

Pathology results are shown in Figure 1.

In CY-treated animals (Figure 1–5, 1–6) islets of viable polymorphic cells were surrounded by large areas of ischemic necrosis, without sign of muscle fibre integrity. No area with viable S180 cells was found, and some areas of granulation tissue with formation of fibrous tissues were found.

In the ASMq 4 g/kg group, ischemic necrosis is obvious as well as phlogocyte infiltrates (Figure 1-11 to 1–12). The ASMq 8 g/kg group also have obvious ischemic necrosis, but less than the 4 g/kg group, and some other areas of granulation tissue were observed (Figure 1-7 to 1–10). The lower ASMq dosage group did not have signs or areas of ischemic necrosis (Figure 1-13 to 1–14).

### Splenic lymphocyte function

**Figure 2:**
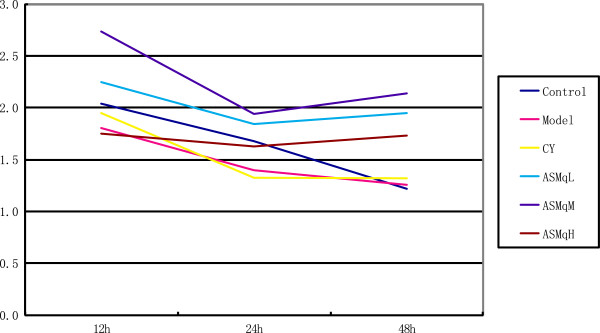
**Splenic lymphocyte proliferation.** Variations in splenic lymphocyte proliferation: a: normal untreated controls; b: model untreated controls injected with S180 cells; c: cyclophosphamide-treated tumour-bearing mice; d, e, f: ASMq-treated tumour-bearing mice.

IN ASMq 2 g/kg, 4 g/kg, 8 g/kg groups splenic lymphocyte proliferation was found, with an apparent dose-effect relationship (Figure 2). At the different times (12 h, 24 h, 48 h) after administration, the 4 g/kg dose was associated with the most T cell proliferation.

## Discussion

ASMq is widely used in traditional Uyghur medicine for the treatment of complex diseases such as tumours. Previous studies have indeed found an antiproliferative effect of ASMq both *in vitro* on HepG2 and CACO cell lines [6], and *in vivo* on methylhydrazine induced colon cancer in rats [2]. It induces cytotoxicity and apoptosis *in vitro*[5,7]. ASMq also appears to have immune-modulating effects [8], which may also play a role in its antiproliferative activity *in vivo.*

In the present study in S180 implanted mice, we confirm the antiproliferative effect of ASMq on the cancer cells. There was an increased spleen index and lymphocyte proliferations. ASMq increased IL-1β, IL-2, TNF-α. IL-1β and IL-2 are important lymphokines in vivo, and can promote the proliferation of T cells, B cells, and macrophages. In the anti-tumour immunity which is predominantly cellular, T cells are the main immune cells that can be cytotoxic directly, or indirectly through the secretion of cytokines TNF-α, IFN-γ. Many of the cytokines , such as IFN-γ or IL-2, can activate natural Killer (NK) cells. NK cells in the absence of antibodies can kill tumour cells in vivo. Activation of NK cells induces LAK cells and cytotoxic T cell maturation, etc., which in the anti-tumour immunity play an important role in regulating lymphocyte proliferation. In addition, activated NK cells can release cytokines TNF-α, IL-2, etc., and stimulate macrophages.

Though these effects were for the most part statistically significant, there was no clear dose-effect relationship, with the middle dose of ASMq often more effective than the higher dose. This may be related to increased cytotoxicity with the higher dose. The antitumoral effects of ASMq were not very different from those of Cyclophosphamide on the S180 model, [10] though the effects on cytokines were different with a greater effect of ASMq on TNF-α and IL2, but not on T4 or T8 distribution.

According to the Chinese cancer research standard, a Chinese herbal medicine is deemed effective when the tumour inhibitory rate reaches 30% and above. ASMq easily surpassesd that threshold at all tested doses [11,12].

Beyond the antitumoral drugs derived from plants (such as the vinca minor derivatives or the taxanes) other traditional herbal medicines have shown antiproliferative or cytotoxic effects on the same S180 mouse model, alone [13] or combined with cyclophosphamide [14], and on other animal tumour models [11]. Other herbal extracts or traditional medicines have demonstrated immune-modulating effects [15,16].

## Conclusions

In conclusion, the Uyghur medicine Abnormal Savda Munziq, that is traditionally used to treat or prevent cancer, showed in this study inhibition of injected S180 myosarcoma in mice. ASMq appeared to activate tumour-inhibiting or killing processes through the activation of lymphocytes and lymphokines or chemokines, in addition to effects on cell division, DNA synthesis and apoptosis demonstrated in vivo on other cancer models [5-7].

## Competing interests

HU is the owner of the patent for the ASMq preparation used here.

None of the other authors declare any conflict of interest.

## Authors’ contributions

Study design was by HU, AY. Experimental work was done by AA, under the supervision of AY, HU, AU, as part of his PhD Thesis. First draft was of paper was written by AA, reviewed and translated from the Chinese by AU. BB and NM reviewed the methodology and results, and rewrote the paper. All authors reviewed and approved the final version.

## Pre-publication history

The pre-publication history for this paper can be accessed here:

http://www.biomedcentral.com/1472-6882/12/157/prepub
